# Compassionate use of roxadustat for treatment of refractory renal anemia in an infant

**DOI:** 10.1007/s00467-023-06240-1

**Published:** 2023-12-13

**Authors:** Yan Yang, Yan Chen, Yang Yang, Haitao Bai, Bizi He, Dengli Liu

**Affiliations:** 1 Department of Pediatrics, The First Affiliated Hospital of Xiamen University, Xiamen, Fujian China; 2 Pediatric Key Laboratory of Xiamen, Xiamen, Fujian China; 3 Institute of Pediatrics, School of Medicine, Xiamen University, Xiamen, Fujian China

**Keywords:** Anemia, Roxadustat, Chronic kidney disease, Infant, Hypoxia-inducible factor prolyl hydroxylase inhibitor, Erythropoiesis-stimulating agent

## Abstract

**Background:**

Erythropoiesis-stimulating agents (ESAs) have played an important role in the treatment of renal anemia in children, but cannot improve hemoglobin to target level in some cases. Roxadustat, a hypoxia-inducible factor prolyl hydroxylase inhibitor, can stimulate endogenous erythropoietin production and regulate iron metabolism even in patients with kidney failure. However, roxadustat has not yet been approved for use in children.

**Case–diagnosis/Treatment:**

We report a case of refractory renal anemia in an 80-day-old boy, who was hyporesponsive to ESAs even in combination with iron supplementation and transfusion. Compassionate use of roxadustat successfully corrected the intractable anemia. Hyperkalemia is a manageable adverse event of concern during follow-up.

**Conclusion:**

The successful experience in this case may inform the clinical utility of roxadustat for refractory renal anemia in children, which should be further confirmed by well-designed prospective clinical trials.

**Supplementary Information:**

The online version contains supplementary material available at 10.1007/s00467-023-06240-1.

## Introduction

Anemia is one of the common clinical manifestations of chronic kidney disease (CKD) in children. Erythropoiesis-stimulating agents (ESAs) have been used to manage renal anemia since the 1980s. Cumulative evidence has proved that ESAs can significantly improve renal anemia and reduce blood transfusion and human leukocyte antigen (HLA) sensitization in children [1]. However, a large proportion of anemic CKD patients do not meet the hemoglobin (Hb) targets even after standard ESA treatment. Furthermore, 10–15% of the chronic dialysis population is hyporesponsive to ESAs. It is reported that iron deficiency and inflammation, secondary hyperparathyroidism, inadequate dialysis, younger age, malnutrition, concomitant medications, and erythropoietin resistance were significantly associated with ESA hyporesponsiveness [2]. New approaches are needed to address the issue of refractory renal anemia in children. Roxadustat (also known as FG-4592) is the first hypoxia-inducible factor-prolyl hydroxylase inhibitor (HIF-PHI) available for clinical use. It is effective in correcting ESA-hyporesponsive anemia in patients on peritoneal dialysis [3].

Unlike ESA, which only regulate the EPO pathway to promote erythropoiesis, hypoxia inducible factor (HIF) stimulates erythropoiesis through a comprehensive and efficient mechanism. It not only promotes the expression of EPO/ EPO receptor (EPOR), but also up-regulates the levels of divalent metal transporter 1 (DMT1) and duodenal cytochrome b (DCytB) to increase the absorption of iron in the intestine, up-regulates the level of transferrin to promote iron transport to the whole body, and up-regulates the level of transferrin receptor (TfR) to increase the uptake of iron by red blood cells, but down-regulates the level of hepcidin to promote the absorption and reuse of iron [4]. HIF is under the control of HIF-prolyl 46 hydroxylases (HIF-PHs) which lead to its degradation. In the situation of hypoxia, HIF-PHs are suppressed, leading to activation of the HIF pathway. Accordingly, HIF-PHI has become an attractive therapeutic target in patients with renal anemia.

However, roxadustat is not approved for any indication in children at present. The effectiveness and safety of roxadustat in infants with renal anemia is not clear. Here, we report on an 80-day-old infant who had CKD stage 5 and renal anemia and showed poor response to ESA treatment. Oral roxadustat treatment effectively improved the hemoglobin level and avoided red blood cell (RBC) transfusion. It is a valuable clue for pediatricians to explore the utility of roxadustat for renal anemia in children.

## Case diagnosis and treatment

**Fig. 1:**
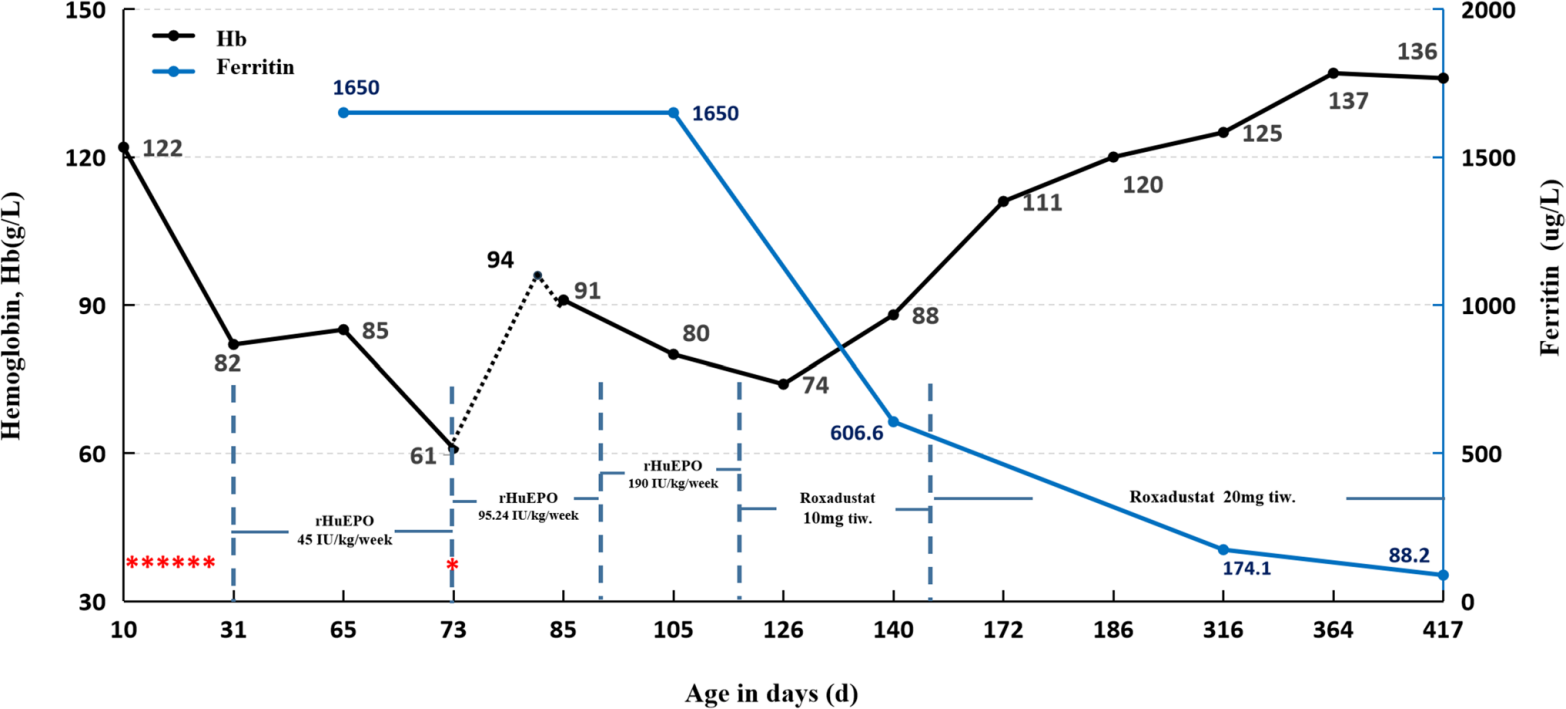
Changes of hemoglobin and serum ferritin during rHuEPO and roxadustat treatment. The black dotted line indicates hemoglobin change after blood transfusion rHuEPO, recombinant human erythropoietin; tiw, three times a week. *, number of transfusions

An 80-day-old boy was admitted to the Department of Nephrology due to pallor for more than 2 months. The infant was delivered naturally at 38^+4^ weeks of gestation (gravida 4 para 2), and his birth weight was 2.79 kg. Bloody amniotic fluid was observed. The placenta and umbilical cord were normal. His skin color was pale associated with poor response and weak crying after birth. The Apgar score was 6 (1 point for heart rate and muscle tone each, 0 point for skin color) at 1 min and 8 points (0 point for skin color) at 5 and 10 min. The infant developed cardiac arrest on the same day. The rescue was successful after cardiopulmonary resuscitation and epinephrine injection. However, oliguria and increasing edema were observed the next day associated with high creatinine level up to 621 μmol/L and urea up to 29.86 mmol/L. His kidneys were generally normal in size. Ten days after birth, ultrasonography showed right kidney 5.1 × 2.4 × 2.8 cm and left kidney 5.4 × 2.8 × 3.1 cm. The edema disappeared after peritoneal dialysis. The urine volume was 150–220 mL/d. Peritoneal dialysis was discontinued due to family factors. His serum creatinine stayed at a high level—about 350 μmol/L. During this period, the boy received comprehensive anti-anemic treatment, including iron supplements, leukocyte-depleted RBC transfusion (3 units in total), and rHuEPO (45 IU/kg/week for 6 weeks), but failed to reach the Hb target. In the first month after birth, the Hb level decreased from 122 to 82 g/L, and further reduced to 61 g/L (HCT 0.191 L/L) in the second month after birth, just before his admission to the Department of Nephrology when he was 80 days old (Fig. 1).

The boy’s parents did not have a family history of kidney disease or consanguinity. His mother had induced abortion twice. The patient has a 21-month-old sister who is healthy. Physical examination on admission found that the infant had the appearance of malnutrition (length 52 cm, weight 4.2 kg, body mass index 15.5 kg/m^2^) and anemia. Laboratory tests showed Hb 61 g/L (HCT 0.191 L/L) reticulocyte percentage 1.79%, absolute reticulocyte count 0.037 × 10^9^/L, hypersensitive C-reactive protein < 0.5 mg/L, serum ferritin > 1650 μg/L (reference range: 10–291 μg/L), and serum parathyroid hormone level 362 pg/mL (reference range 7–53 pg/mL). The serum folic acid (32.1 ng/mL) and vitamin B12 (996 pg/mL) levels were normal. Blood gas analysis was generally normal. Other laboratory tests showed serum albumin 37.2 g/L, phosphorus 2.14 mmol/L, calcium 2.15 mmol/L, potassium 5.17 mmol/L, cystatin C 6.83 mg/L, BUN 20.06 mmol/L, and Cr 368 μmol/L.

After admission, the background treatment included iron supplements, vitamin B12, rHuEPO, calcitriol, and calcium agent for hyperparathyroidism. The Hb level recovered to 94 g/L (HCT 0.293 L/L) after transfusion of RBC (0.5 unit). After rHuEPO dose was increased to 95.24 IU/kg/week for 2 weeks, followed by 190 IU/kg/week for 4 weeks, the anemia was still not corrected (Figure 1). Increased dosage of rHuEPO injection also caused intolerable pain. The boy’s parents lacked healthcare knowledge and nursing experience, it was a long distance from the patient’s residence to the available healthcare facility, and COVID-19 pandemic restrictions made rHuEPO treatment inconvenient. Oral roxadustat was considered a reasonable option for the boy’s intractable anemia. After discussion between the parents of the patient, the clinicians, and the representative of the drug manufacturer, an agreement was reached to accept roxadustat for compassionate use. After approval by the Ethics Committee of the First Affiliated Hospital of Xiamen University, the treatment was then switched from rHuEPO to oral roxadustat 10 mg three times a week at 4 months of age when Hb was 74 g/L. After 4 weeks of treatment, Hb was 75–88 g/L. Roxadustat dose was adjusted to 20 mg three times a week. Hb level increased gradually up to 111 g/L 4 weeks later. Currently, the Hb level is maintained at about 120 g/L under roxadustat treatment at current dose (Supplementary Table 1).

The patient has been followed up for more than 2 years. Comprehensive treatment has improved the acidosis, hyperparathyroidism, growth, and development of the patient. However, the kidney function did not improve, which was still at CKD stage 5 (Cr 214 μmol/L). The last follow-up ultrasound on June 12, 2023, revealed shrinkage of kidneys (right kidney 4.7 × 2.0 × 2.8 cm and left kidney 4.8 × 3.1 × 2.4 cm), consistent with CKD changes. The patient developed CKD in the neonatal period which might be due to perinatal events such as perinatal asphyxia and cardiac arrest. In addition, genetic factors should also be considered because the baby developed acute kidney disease (AKD) and CKD even after active treatment. During roxadustat treatment, no serious adverse events were identified by monitoring liver and kidney function, serum electrolytes, blood pressure, and electrocardiogram. Serum potassium increased slightly, ranging from 4.47 to 6.00 mmol/L. Oral furosemide (0.5 mg/kg, three times a day) treatment could keep the serum potassium level below the upper normal limit. More details about switching rHuEPO to roxadustat for anti-anemia therapy and the corresponding changes of Hb are provided in Supplementary Table 1.

## Discussion and conclusion

Renal anemia not only affects the quality of life of patients [5], but also contributes to the progression of kidney disease, and increases the risk of cardiovascular events and death [1, 6]. The treatment of renal anemia in children is very challenging, especially when the anemia is refractory and hyporesponsive to ESAs. According to the data of the North American Pediatric Renal Trials and Collaborative Studies (NAPRTCS) [7], Hb level stayed lower than the normal range in more than 20% of stage 4 CKD patients and more than 40% of stage 5 CKD patients in children receiving ESA treatment. The data of the European Dialysis and Transplant Association in 2012 [8] showed that Hb level did not reach the Hb target in 33.4% of children below 2 years and 31.2% of those above 2 years who were on dialysis. This will lead to an attempt to increase the dose of ESAs to reach the Hb target. In some observational studies [9, 10], it is interesting to note that even a higher dose of ESAs could not increase the rate to reach the Hb target in infants. ESA hyporesponsiveness and the safety concerns of high-dose ESAs urge clinicians to find new strategies to address renal anemia.

Roxadustat is a small molecule compound, which can reversibly inhibit the activity of prolyl hydroxylase domain (PHD), mimic the hypoxic environment in the body, and induce stable expression of HIF in a transient and dose-dependent manner, thus promoting the expression of the target gene *EPO* in the downstream HIF, and improve renal anemia by inducing erythropoiesis. In addition, roxadustat can also promote the expression of EPOR, reduce the level of hepcidin, increase the absorption, transport and utilization of iron, and so promote the formation of red blood cells via actions on multiple targets [4]. Roxadustat was firstly approved in China in 2018 for treatment of anemia in CKD patients based on the results of a pivotal phase 3 clinical trial [3]. Clinical trials of HIF-PHI have shown that these agents could ameliorate functional iron deficiency and increase Hb levels in CKD patients with anemia [11]. It has been suggested that HIF-PHI is also effective in patients with ESA hyporesponsiveness, such as high-level inflammation and secondary hyperparathyroidism [12].

Nevertheless, roxadustat has not been approved for any indication in children. The results of Study NCT04925011 [13] are expected to expand the indications of roxadustat to children. The patient in this report is the youngest one as far as we know whose renal anemia was successfully treated with roxadustat. ESA treatment did not increase Hb satisfactorily for this boy even after increasing the dose of ESA. The poor effect and inconvenience of administration of high-dose ESA allowed the clinician to switch therapy from ESA to roxadustat. The patient developed slight hyperkalemia during roxadustat treatment, which was consistent with the safety profile of roxadustat [14]. The adverse event was probably due to high-potassium diet or CKD itself. It was manageable by furosemide treatment, which could keep the serum potassium level below the upper normal limit. Other adverse events of HIF-PHI in adults such as hypertension, cardiovascular events, retinopathy, tumors, gastrointestinal symptoms, thromboembolic events, seizure, and severe infections [14, 15] were not observed in this infant. We will follow up with the patient to determine the risk of these adverse events.

The successful treatment of refractory renal anemia by compassionate use of roxadustat in this case suggests that oral roxadustat may be favorable for renal anemia in case of poor response to ESAs. However, this single case is only a clue rather than convincing evidence for clinicians to consider the clinical use of roxadustat in children. Well-designed prospective clinical trials are still needed to confirm the real efficacy and safety of roxadustat in children.

### Supplementary Information

Below is the link to the electronic supplementary material.

Supplementary file1 (DOCX 15 KB)

## Data Availability

Relevant data are available on reasonable request.
