# Supplementary material for: Compassionate use of roxadustat for treatment of refractory renal anemia in an infant
Source: Pediatr Nephrol. 2023 Dec 13;39(3):911–4. doi: 10.1007/s00467-023-06240-1 (PMC10817834; doi:10.1007/s00467-023-06240-1)
Supplement: Supplementary file 1 — Supplementary file1 (DOCX 15 KB) [file 467_2023_6240_MOESM1_ESM.docx]

**Supplementary Table 1 Changing results of laboratory tests after roxadustat treatment**

| Age (days) | HB  (g/L) | HCT  (L/L) | FER  (μg/L) | TSAT  (%) | ALB  (g/L) | Cr  (μmol/L) | K+  (mmol/L) | iPTH  (pg/mL) |
| --- | --- | --- | --- | --- | --- | --- | --- | --- |
| 105 | 80 | 0.240 | 1650.0 | 85.51 | 47.1 | 335 | 5.1 | 362 |
| 316 | 125 | 0.398 | 174.1 | 28.65 | 44.7 | 157 | 5.2 | 191 |
| 417 | 136 | 0.447 | 88.2 | 35.29 | 45.6 | 182 | 4.85 | 144 |
| 550 | 141 | 0.425 | NA | NA | 45.4 | 216 | 4.49 | 374 |
| 886 | 126 | 0.380 | NA | NA | 43.7 | 214 | 4.32 | 203 |

HB, hemoglobin; HCT, hematocrit; FER, ferritin; TSAT, transferrin saturation; ALB, albumin; Cr, creatinine; K+, potassium; iPTH, intact parathyroid hormone
